# *Phlebotomus sergenti* in a Cutaneous Leishmaniasis Focus in Azilal Province (High Atlas, Morocco): Molecular Detection and Genotyping of *Leishmania tropica*, and Feeding Behavior

**DOI:** 10.1371/journal.pntd.0003687

**Published:** 2015-03-31

**Authors:** Malika Ajaoud, Nargys Es-Sette, Rémi N Charrel, Abderahmane Laamrani-Idrissi, Haddou Nhammi, Myriam Riyad, Meryem Lemrani

**Affiliations:** 1 Laboratoire de Parasitologie et Maladies Vectorielles, Institut Pasteur du Maroc, Casablanca, Morocco; 2 Centre d’Etudes Doctorales des Sciences de la Santé, Faculté de Médecine et Pharmacie, Casablanca, Morocco; 3 Aix Marseille University, IRD French Institute of Research for Development, EHESP French School of Public Health, EPV UMR_D 190 "Emergence des Pathologies Virales", & IHU Méditerranée Infection, APHM Public Hospitals of Marseille, Marseille, France; 4 Service de Parasitologie, Direction d'Epidémiologie et de Lutte contre les Maladies, Ministère de la Santé, Rabat, Morocco; 5 Equipe de Recherche sur les Leishmanioses Cutanées, Faculté de Médecine et Pharmacie, Casablanca, Morocco; The Faculty of Medicine, The Hebrew University of Jerusalem, ISRAEL

## Abstract

**Background:**

*Phlebotomus* (*Paraphlebotomus*) *sergenti* is at least one of the confirmed vectors for the transmission of cutaneous leishmaniasis caused by *Leishmania tropica* and distributed widely in Morocco. This form of leishmaniasis is considered largely as anthroponotic, although dogs were found infected with *Leishmania tropica*, suggestive of zoonosis in some rural areas.

**Methodology and Findings:**

This survey aimed at (i) studying the presence of *Leishmania* in field caught *Phlebotomus sergenti*, (ii) investigating genetic diversity within *Leishmania tropica* and (iii) identifying the host-blood feeding preferences of *Phlebotomus sergenti*. A total of 4,407 sand flies were collected in three rural areas of Azilal province, using CDC miniature light traps. Samples collected were found to consist of 13 species: *Phlebotomus* spp. and 3 *Sergentomyia* spp. The most abundant species was *Phlebotomus sergenti*, accounting for 45.75 % of the total. 965 female *Phlebotomus sergenti* were screened for the presence of *Leishmania* by ITS1-PCR-RFLP, giving a positive rate of 5.7% (55/965), all being identified as *Leishmania tropica*. Nucleotide heterogeneity of PCR-amplified ITS1-5.8S rRNA gene-ITS2 was noted. Analyses of 31 sequences obtained segregated them into 16 haplotypes, of which 7 contain superimposed peaks at certain nucleotide positions, suggestive of heterozygosity. *Phlebotomus sergenti* collected were found to feed on a large variety of vertebrate hosts, as determined by *Cytochrome b* sequencing of the DNA from the blood meals of 64 engorged females.

**Conclusion:**

Our findings supported the notion that *Phlebotomus sergenti* is the primary vector of *Leishmania tropica* in this focus, and that the latter is genetically very heterogeneous. Furthermore, our results might be suggestive of a certain level of heterozygosity in *Leishmania tropica* population. This finding, as well as the feeding of the vectors on different animals are of interest for further investigation.

## Author Summary

In Morocco three *Leishmania* species have been reported to cause cutaneous leishmaniasis: *Leishmania major*, *Leishmania tropica* and less frequently *Leishmania infantum*. Amongst these clinically important *Leishmania* species, *Leishmania tropica* is considered as a public health problem by the Ministry of Health in Morocco and other endemic countries. *Phlebotomus sergenti* is the known vector, which is thought to take blood meals mainly from humans, since they appear to be the sole reservoir, considering anthroponosis of the cutaneous leishmaniasis caused by *L*. *tropica* in many endemic areas. In the present study, we investigated by molecular tools the presence of *Leishmania* in field caught *Phlebotomus*, as well as the heterogeneity of *Leishmania tropica* in a cutaneous leishmaniasis focus in High Atlas of Morocco. Our results showed a high infection rate of *Phlebotomus sergenti*, which may be a consequence of high level of the parasite circulating in this region; and underlined the important genetic heterogeneity of *Leishmania tropica* in Morocco. Analysis of the blood meals of the vectors showed that *Phlebotomus sergenti* fed on a variety of vertebrates, including wild animals, such as rodent, monkey and bat. Whether these animals play any role in the maintenance of *Leishmania tropica* in this focus awaits further investigation.

## Introduction

Leishmaniases are complex diseases of worldwide distribution caused by >20 *Leishmania* species, which are parasitic protozoa transmitted by the bite of infected female sand flies. The disease affects 98 Mediterranean and other endemic countries putting a population of 350 million people at risk of infection and causing 1.3 million new cases and 20,000 to 30,000 deaths annually [1, 2].

In Morocco, leishmaniasis is a public health problem, with 8,862 cases notified in 2010 alone. It is widely distributed from the mountains of the Riff to the peri-arid foothills of the Anti-Atlas [3, 4]. Three *Leishmania* species are responsible for cutaneous leishmaniasis (CL) in Morocco: *L*. *major*, *L*. *tropica* and *L*. *infantum*. CL due to *L*. *tropica* is endemic in the semi-arid regions and the sand fly species *Phlebotomus* (*Paraphlebotomus*) *sergenti* Parrot is considered to be the vector [5, 6]. This form of CL was described for the first time in Tanant, a rural locality in Azilal province in High Atlas [7]. Thereafter, a large rural focus has been further identified in the centre and the south of Morocco [6]. In the mid 1990s, CL caused by *L*. *tropica* was then reported in Taza province, in northern Morocco [8] and early in 2000, outbreaks occurred in emerging foci in the central and northern parts of the country, and *L*. *tropica* was also even found in areas previously only known as *L*. *major* foci [9, 10]. CL due to *L*. *tropica* is basically anthroponotic, although the parasite was also isolated from dogs in some leishmaniasis foci in Morocco, suggesting that *L*. *tropica* could also be zoonotic at least in some areas [5, 11, 12].

Blood meal analysis of haematophagous insects has been informative in elucidating their natural host-feeding preference or host-feeding pattern for identifying potential reservoirs [13]. For sand flies, the blood meal sources have been identified initially by serological techniques, like ELISA [14, 15], agarose gel diffusion [16], counter immunoelectrophoresis [17], precipitin test [18, 19] and a more laborious histological technique [20]. Although all these methods have been useful in identifying vertebrate hosts for many haematophagous insects, they lack sensitivity and are time-consuming.

In the last decade, several PCR-based molecular approaches have been developed to enhance the specificity of insect blood meal identification [21, 22]. Analysis of blood meal sources of the engorged vectors has been greatly facilitated by the increasing availability of animal genomic database at the genus and species levels (NCBI, EBI…). Downstream applications of PCR based on primers designed from multiple alignments of the mitochondrial *Cytochrome b* gene of different vertebrate species have identified avian and mammalian blood sources in various species of sand flies. The sequencing of *Cyt b* gene, as well as the *Cyt b*-PCR-RFLP and the *Cyt b*-PCR combined with reverse line blot have been used to identify the blood meals sources of sand flies [23–25].

The vector incrimination is classically based on the dissection of freshly caught individual sand flies and subsequent culture of parasites present in the gut [26]. This method needs dissecting expertise and is time consuming, since the *Leishmania* infection rate in sand flies is usually very low [27]. In recent years, several molecular techniques have been developed to identify *Leishmania* infection in infected phlebotomine sand flies. Because of excellent sensitivity and specificity, these methods have the potential to challenge the time-consuming isoenzyme technique for timely characterization of *Leishmania* strains. They also play a prominent role in epidemiological prospective surveillance activities which are critical for planning targeted control measures of leishmaniasis [15, 28, 29].

In the present study, we used molecular tools (ITS1-PCR-RFLP) to detect and identify *Leishmania* species within naturally infected sand flies collected from three rural localities of Azilal province in Morocco. We also applied nested-PCR of *Leishmania* ITS-rDNA genes for sequencing analyses to characterize *L*. *tropica*. The blood meals of engorged flies were also analyzed to determine the putative animal reservoirs of *L*. *tropica*, using a PCR technique based on the C*yt b* gene of vertebrate mitochondrial DNA (mtDNA).

## Materials and Methods

### Study sites

**Fig 1:**
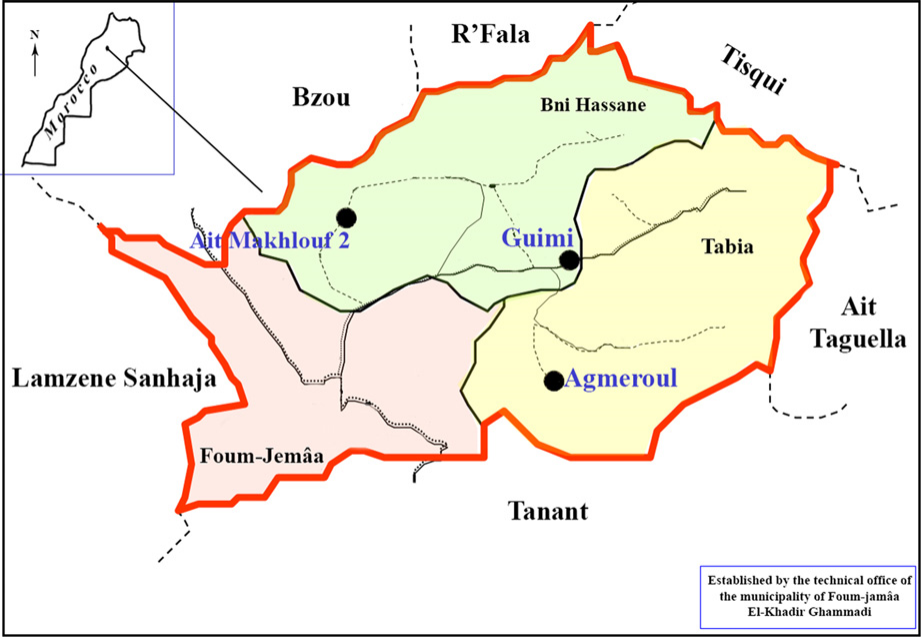
Location of the three sampling areas (Ait Makhlouf 2, Agmeroul and Guimi) in Azilal province.

The sand flies were collected in three neighboring rural areas in the province of Azilal, High Atlas of Morocco. The three regions are: Ait Makhlouf II: 31°01’23”N, 6°58’53”O and Guimi: 32°00’12”N, 6°55’03”O, which are located in Beni Hassan sector, and Agmeroul: 31°58’38”N, 6°51’12”O in Tabia sector. These localities are at different altitude from 679 m to 840 m, separated from one another at a distance of 5 to 11 km (Fig. 1). Vegetation is sparse and mainly dominated by cactus, jujube plant, olive, and almond trees. Agriculture remains the primary source of income and is mainly based on the production of wheat, almonds and olives. Livestock include chicken, sheep, cattle and horses and most homes have at least one dog. Houses are mainly of the traditional type, constructed with adobe. The villages are surrounded by mountains, with some groundwater sources used for irrigation.

### Collection, dissection and identification of sand flies

Phlebotomine sand flies were caught during three consecutive nights from June to October 2011, using CDC light traps placed inside the houses and in domestic animal shelters, in the three rural sites. The traps placed about 1.5 meter above the ground were set before sunset and collected the next morning. The collected sand flies were then placed in 1.5 mL Eppendorf tubes, transferred to the lab in dry ice and kept at -80°C. All the sand flies were washed and dissected. The head and genitalia were used for morphological identification using Moroccan morphologic key [4]. To distinguish *P*. *perniciosus* and *P*. *longicuspis* a very closely related species, we used the morphological criteria updated by Benabdennbi *et al*. (1999) [30] and Guernaoui *et al*. (2005) [31] based on the morphological features of genitalia and the number of coxite hairs. *P*. *perniciosus* is characterized by 2 aedeagi forms: (i) copulatory valve with apex bifid; and (ii) copulatory valve with curved apex, the number of coxite hairs being up to 14. *P*. *longicuspis* has copulatory valve ending with a single and long point, and 19 or more coxite hairs.

The remainder of the body of engorged and unengorged females after dissection was stored sterilely in 1.5 mL microtube at -20°C until use.

### DNA extraction

Genomic DNAs were extracted from unengorged and engorged female *P*. *sergenti* sand fly by using the Kit PureLink™ Genomic DNA Mini Kit and the QIAamp® DNA Blood Mini Kit, respectively, according to the manufacturer’s instruction.

### Molecular detection and identification of *Leishmania* species in *P*. *sergenti* females

For *P*. *sergenti* females, the leishmanial ribosomal internal transcribed spacer 1 (ITS1) region was amplified, using the primers LITSR (5′-CTGGATCATTTTCCGATG-3′) and L5.8S (5′-TGATACCACTTATCGCACTT-3′), following the protocol described by Schonian *et al*. (2003). This PCR was used to amplify a 300 to 350 bp fragment; then the ITS1-PCR products were digested with restriction endonuclease *Hae*III enzyme for *Leishmania* species identification [32]. The restriction profiles were analyzed by electrophoresis on 3% agarose gel containing ethidium bromide, and compared with *Leishmania* reference strains: *L*. *major* (MHOM/SU/73/5ASKH), *L*. *tropica* (MHOM/SU/74/K27) and *L*. *infantum* (MHOM/TN/80/IPT1). A 100 bp DNA size marker was used. Negative controls (containing water, without DNA) were added to each PCR run.

Further identification of *Leishmania* parasites was done using nested PCR amplification of ITS1-5.8S rRNA gene-ITS2. The primers IR1 and IR2 were used for the first PCR stage and ITS1F and ITS2R4 for the second stage, as previously described by Parvizi and Ready [33]. Negative controls were included to each PCR run. All PCR products were analyzed by electrophoresis on 1% agarose gel containing ethidium bromide. We used the standard DNA fragment 100 bp ladder as a size marker. The nested-PCR products (~460 bp including primers) were sequenced, using the primers ITS1F and ITS2R4. The sequences obtained were edited using Seq Scape, BioEdit softwares and aligned using ClustalW in MEGA6 software.

A phylogenetic tree was constructed by using the Maximum Likelihood method based on the Tamura-Nei model. Initial tree(s) for the heuristic search were obtained automatically by applying the Maximum Parsimony method. Bootstrap replicates were performed to estimate the node reliability, and values were obtained from 1,000 randomly selected samples of the aligned sequence data. Sequences were compared with entries retrieved from GenBank. Evolutionary analyses were conducted in MEGA6.

### Molecular identification of *P*. *sergenti* females

Morphologically identified *P*. *sergenti* females were confirmed by sequence analyses of PCR-amplified mitochondrial DNA fragment encompassing *Cyt b* region using PhleF (5’-AAT AAA TTA GGA GTA ATT GC-3’) and PhleR (5’-GCC TCG AWT TCG WTT ATG ATA AAT T-3’) primers [34]. A 500 bp fragment was PCR-amplified using the following reagents: 1X PCR buffer, 2.5 mM MgCl_2_, 0.4 mM primers (F and R), 0.2 mM dNTPs, 4 μL DNA, made up with distilled water to a final volume of 25 μL for each reaction. The PCR runs were each initiated as follows: 94°C for 12 min, followed by 5 cycles of 94°C for 30 s; 52°C for 30 s, 72°C for 1 min. The PCR reaction was then continued for 30 cycles under the same conditions, except for the annealing step (54°C for 1 min) and a final extension at 72°C for 10 min. The PCR products were sequenced, employing the same primers used for the PCR. Sequences were processed and aligned, using the multiple alignment programs Seq Scape and BioEdit.

A phylogenetic tree was constructed by using the Neighbor-Joining method, in agreement with Kimura 2-parameter model, at uniform rate for transitions and transversions. Bootstrap replicates were performed to estimate the node reliability, and values were obtained from 1,000 randomly selected samples of the aligned sequence data. Sequences were compared with entries retrieved from GenBank. The phylogenetic tree was constructed by using the MEGA6 software.

### Blood meal identification

A region of *Cyt b* gene from host mtDNA was amplified for blood meal source identification of the blood-fed female specimens. The assay is based on specific amplification and sequencing of the blood meal–derived the region of the host mtDNA *Cyt b* gene. Modified vertebrate-universal specific primers L14841 (5’-CCATCCAACATCTCAGCATGATGAAA-3’) and H15149 (5’-CCCCTCAGAATGATATTTGTCCTCA-3’) were used to amplify a 359 bp segment of the *Cyt b* gene [35, 36].

PCR reaction mixture contained 1x PCR Rxn Buffer, 3 mM MgCl_2_, 300 μM deoxynucleotide triphosphates, 0.4 μM of each primer, 1 unit of *Taq* DNA polymerase, 3 μL of DNA template solution and distilled water in a final volume of 25 μL. Samples were incubated at 95°C for 5 min; followed by 36 cycles each at 95°C for 30 s, 60°C for 50 s, and 72°C for 40 s; and a final extension at 72°C for 5 min. A negative control was included for each run. Amplified products were analyzed by electrophoresis on 1% agarose gels stained with 2 mg/mL ethidium bromide and visualized under ultraviolet (UV) light. The amplified products were sequenced on both strands, using a Big Dye Terminator v3.1. Sequences were edited and aligned using Seq-Scape and BioEdit softwares.

The sequences obtained were aligned with those deposited in the GenBank database through the BLAST program for the identification of blood meal vertebrate sources. Sequences of a given pairwise alignment with the lowest E-value were selected as the most likely species of blood meal vertebrate source.

### Biological diversity analysis

In order to characterize the species diversity of sand fly populations, several parameters were used: (i) species richness (S) corresponding to the number of species present in the studied habitat; (ii) relative abundance (*p*_*i*_) reflecting the proportion of individuals belonging to the species *i*.

These parameters were used to calculate different ecological indexes [37, 38].

Simpson’s diversity index (D) that quantifies the biodiversity of a habitat. It takes into account the number of species present as well as the abundance of each species. $D=\frac{1}{\sum_{i=1}^{S}pi^{2}}$

Shannon index (*H’*) that quantifies species diversity: $H^{\prime }=-\sum_{i=1}^{s}p_{i}lnp_{i}$

Pielou index (*J’*): to calculate the equitability of the distribution of species.

*J′* = *H′*/*ln*(*S*) where *H’* is the value of Shannon index and *S* is the species richness.

## Results

### Phlebotomine sand fly fauna

A total of 4,407 sand flies (2,007 females and 2,400 males) were collected in the three rural areas of Azilal province. Morphological analysis identified 10 *Phlebotomus* species of three subgenera as follows: *P*. *sergenti*, *P*. *kazeruni* and *P*. *chabaudi* of the subgenus *Paraphlebotomus*; *P*. *perniciosus*, *P*. *longicuspis*, *P*. *perfiliewi*, *P*. *ariasi*, *P*. *langeroni* and *P*. *chadlii* of the subgenus *Larroussius* and *P*. *papatasi* of the subgenus *Phlebotomus*. Three *Sergentomyia* species were also identified: *Sergentomyia minuta*, *S*. *antennata* and *S*. *fallax*.

The predominant species was *P*. *sergenti;* with a relative abundance of 47.32% of the sand flies belonging to the genus *Phlebotomus* and 45.75% of the total sample.

*P*. *perniciosus* and *P*. *longicuspis* are the next most abundant species, representing 26.82% and 14.23% of the sample respectively (Table 1). July had the highest sand fly abundance, whereas the greatest specific richness (S) was found in September, with the occurrence of eleven species against ten, nine and five species recorded in June, July and October, respectively. The three biodiversity indices were maximal in September (Table 2).

**Table 1:** Species diversity, abundance and relative frequency.

	June			July			September			October			Total		
Species	Abundance		Frequency	Abundance		Frequency	Abundance		Frequency	Abundance		Frequency	Abundance		Frequency
	F	M	(%)	F	M	(%)	F	M	(%)	F	M	(%)	F	M	(%)
***P*. *sergenti***	338	314	40.1	404	572	52.7	139	112	35.01	84	53	64.62	965	1051	45.75
***P*. *perniciosus***	162	408	35.06	94	309	21.76	99	76	24.41	19	15	16.04	374	808	26.82
***P*. *longicuspis***	169	63	14.27	185	103	15.55	53	30	11.58	14	10	11.32	421	206	14.23
***P*. *papatasi***	57	68	7.69	31	28	3.19	46	44	12.55	7	6	6.13	141	146	6.51
***P*. *perfiliewi***	5	-	0.31	-	-	-	-	-	-	-	-	-	5	-	0.11
***P*. *ariasi***	2	-	0.12	-	-	-	1	-	0.14	-	-	-	3	-	0.07
***P*. *chabaudi***	1	-	0.06	1	-	0.05	1	-	0.14	-	-	-	3	-	0.07
***P*. *kazeruni***	-	-	-	-	1	0.05	-	-	-	-	-	-	-	1	0.02
***P*. *langeroni***	-	-	-	-	-	-	-	2	0.28	-	-	-	-	2	0.05
***P*. *chadlii***	-	-	-	-	-	-	-	1	0.14	-	-	-	-	1	0.02
***S*. *minuta***	21	4	1.54	4	2	0.32	38	10	6.69	-	-	-	63	16	1.79
***S*. *antennata***	1	2	0.18	1	4	0.27	3	11	1.95	-	-	-	5	17	0.5
***S*. *fallax***	1	-	0.06	-	2	0.11	23	16	5.44	3	1	1.89	27	19	1.04
**Unidentified**	-	10	0.62	-	111	5.99	-	12	1.67	-	-	-	-	133	3.02
**Total**	**757**	**869**	**100**	**720**	**1132**	**100**	**403**	**314**	**100**	**127**	**85**	**100**	**2007**	**2400**	**100**

**Table 2:** Species diversity of Phlebotomine sand fly, Species richness (S), Shannon-Wiener diversity index (H) and equitability (J’).

Month	NF	NM	N	S	D	H’	J’
June	757	869	1626	10	3.23	1.35	0.59
July	720	1132	1852	9	2.83	1.29	0.59
September	403	314	717	11	4.58	1.75	0.73
October	127	85	212	5	2.18	1.07	0.66

NF: Number of females. NM: Number of males. N: Number of specimens. S: Specific richness. D: Simpson diversity index. H’: Shannon index. J’: Pielou index.

### *Leishmania*-infected flies and parasite typing

#### *Leishmania* ITS1-PCR-RFLP

A total of 965 *P*. *sergenti* females caught by light traps were screened for *Leishmania* infection. 687 unfed specimens collected in June and July were grouped into 35 pools, each one containing up to 20 sand flies. The remaining 278 flies were tested individually for the presence of *Leishmania* DNA using ITS1 PCR-RFLP method. *Leishmania* DNA was amplified from samples of 16 pooled *P*. *sergenti* and 39 individual fly specimens. Altogether, 55 samples were PCR-positive, giving an expected single band of approximately 330 bp (Table 3). Digestion of the amplicons with *Hae*III produced the RFLP-specific pattern of *L*. *tropica* for all the 55 positive samples. The infection rate estimated based on this PCR positivity is 5.69% overall, and 25% and 3.16% for fed and unfed female flies, respectively (Table 3).

**Table 3:** *Leishmania tropica* infection within fed and unfed *Phlebotomus sergenti* females.

	Fed specimens		Unfed specimens		Rate of *L*. *tropica* infection
Month	Individual	*L*. *tropica* infection	Pool* / Individual	*L*. *tropica* infection	
June	9	3	17*	7	10/338 (2.95%)
July	46	10	18*	9	19/404 (4.70%)
September	42	10	97	5	15/139 (10.79%)
October	15	5	69	6	11/84 (13.09%)
Total	112	28 (25%)	853	27 (3.16%)	55/965 (5.69%)

(* = pool)

#### Nested PCR of ITS1-5.8S rRNA gene-ITS2

The 55 *P*. *sergenti* samples positive for *Leishmania* by ITS1-PCR were also positive by the nested PCR for ITS1-5.8S rRNA gene-ITS2 providing a unique 460-bp band. Of 55 PCR products, a total of 31 were completely sequenced in both directions, and showed to correspond to *L*. *tropica*. Sequences alignment showed substantial heterogeneity, and phylogenetic studies segregated them into 16 *L*. *tropica* haplotypes (GenBank accession numbers KM454141-KM454156). The most common haplotype (KM454142) included 14 sequences, presenting 99% of identity with the Indian strain of *L*. *tropica* (FJ948458).

**Fig 2:**
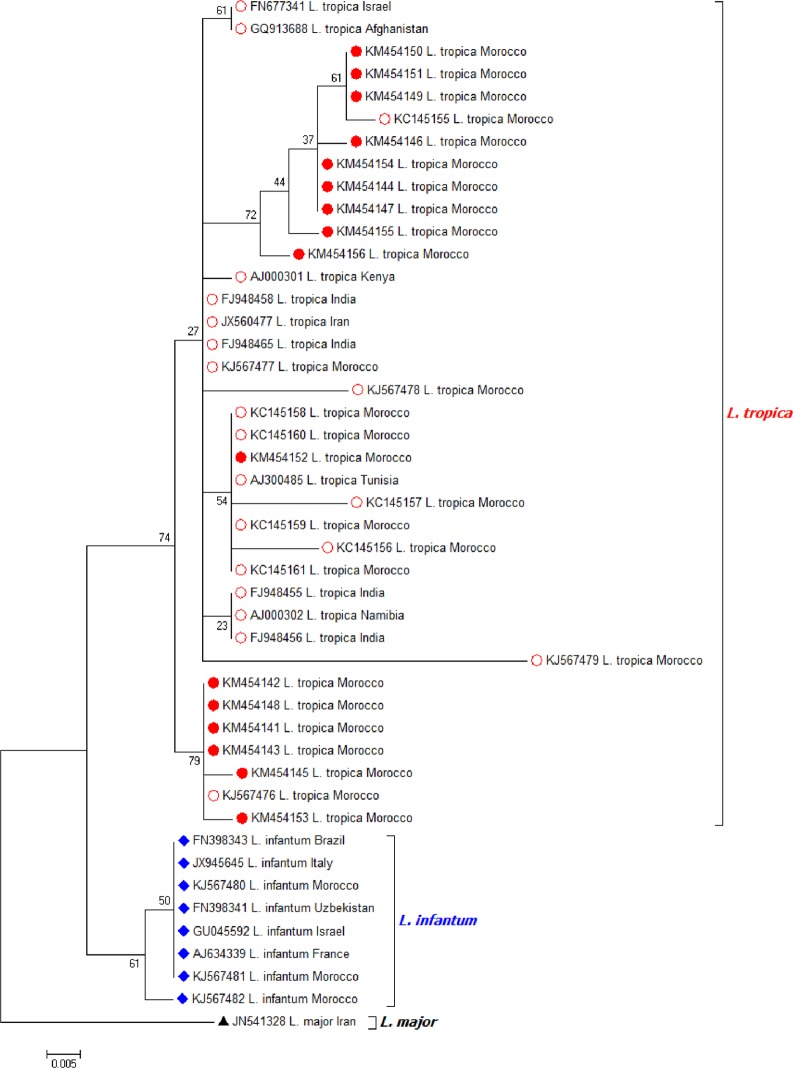
Molecular phylogenetic analysis by Maximum Likelihood method of ITS rDNA sequences of different strains of *Leishmania*. (Red discs: *L*. *tropica* sequences).

Another 16 sequences were grouped into 15 haplotypes: KM454141 and KM454143 to KM454156 highly similar (97% to 99% identity) to *L*. *tropica* strains from India (FJ948456, FJ948455, FJ948465, FJ948458), they differed from the common haplotype by 1 to 18 nucleotides. One of the 15 haplotypes is highly similar to that identified previously in CL patients (KC145159) from an emerging focus of CL in central Morocco (Fig. 2).

**Fig 3:**
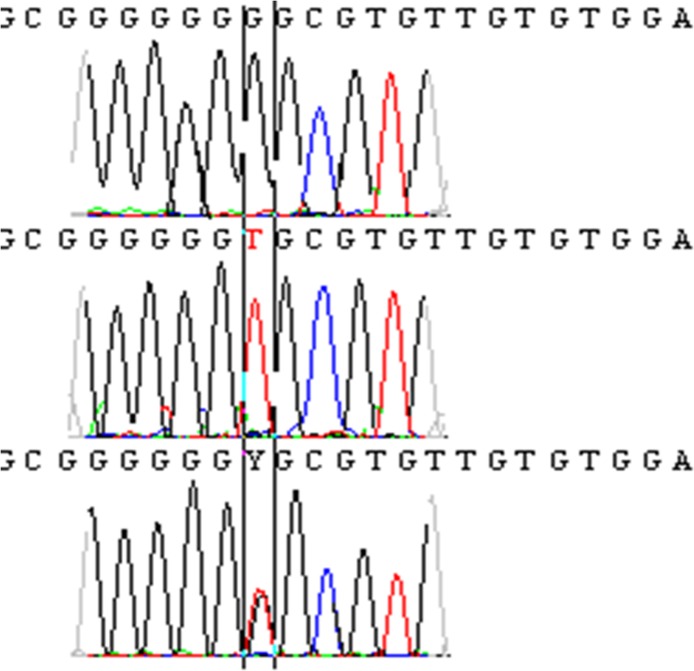
ITS-5.8 rDNA genes sequences with two different bases T and C, and one sequence with two superimposed peaks at the same nucleotide position.

In addition, the analysis of our sequences showed seven sequences with two superimposed peaks, which differ in position from one sequence to another (Fig. 3).

All the other sand fly species identified were pooled mono-specifically and screened for *Leishmania* infection by PCR. None of these samples was PCR-positive, suggesting that *P*. *sergenti* is the only vector in this area.

### Molecular identification of *P*. *sergenti* females

**Fig 4:**
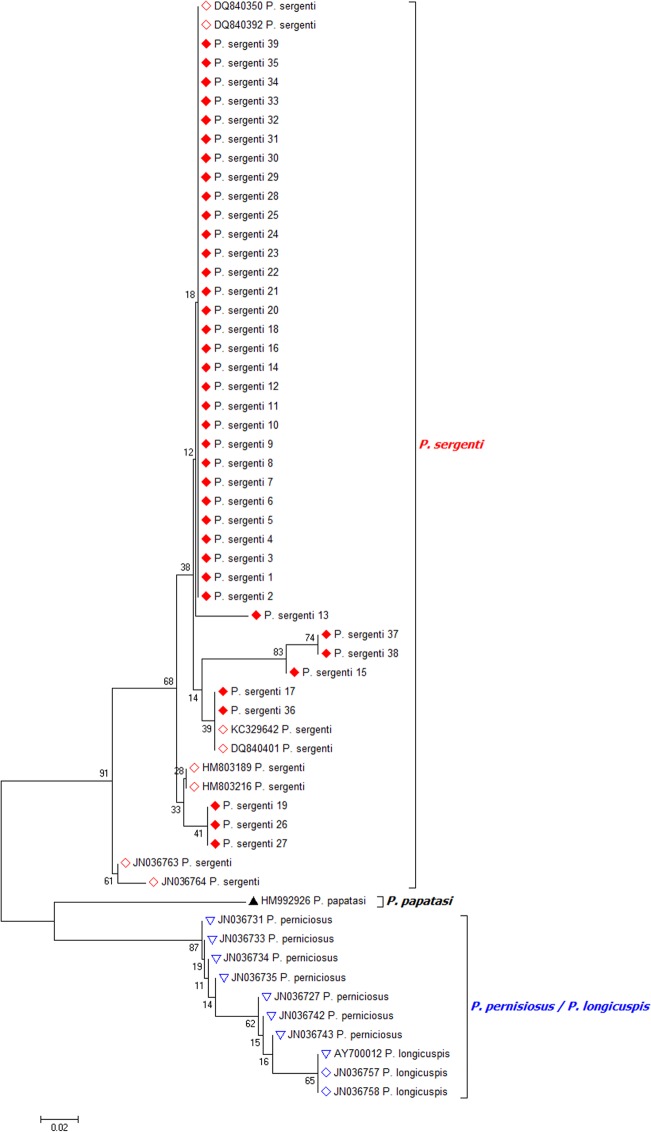
Phylogenetic analysis of the mitochondrial *Cytochrome b* sequences of *P*. *sergenti* and their homologous in GenBank *P*. *sergenti*, *P*. *perniciosus*, *P*. *longicuspis and P*. *papatasi*. (Red lozenges: *L*. *tropica* infected *P*. *sergenti* females).

All the 39 *L*. *tropica* infected female *P*. *sergenti* that were identified morphologically, were validated by molecular characteristics based on the sequencing of *Cyt b* fragment. The alignment of the *Cyt b* sequences obtained confirmed that all samples corresponded to *P*. *sergenti*. All sequences were similar to *P*. *sergenti Cyt b* sequences: DQ840350, JN036763, DQ840392 and JN036764. Phylogenetic analyses grouped them in a distinct clade with other *P*. *sergenti* retrieved from GenBank (Fig. 4).

### Sand fly blood meal identification

One hundred and twelve full or partially blood-fed females (11.6% of the total *P*. *sergenti* females collected) were tested for their blood feeding preferences. The *Cyt b* gene sequencing revealed that 64 *P*. *sergenti* fed on a variety of vertebrate hosts, including humans, chickens (*Gallus gallus*), rodents (*Myomyscus brockmani*, *Mesocritus brandti*, *Dipodomys ordii*: 83–91% of identity), birds, cattle (*Bos Taurus*), rabbit (*Oryctolagus cuniculus*), bat (*Molossus molossus* 89% of identity) and monkey (*Macaca nigra* 95% of identity) (Table 4).

**Table 4:** The blood meal origins of *Phlebotomus sergenti* females identified by *Cyt b* analysis.

	Blood meals source								
	Human	Chicken	Rodent	Bird	Cattle	Rabbit	Monkey	Bat	Total
**Number of *P*. *sergenti***	41	8	7	4	1	1	1	1	**64**
**Infected *P*. *sergenti***	10	3	1	1	-	-	-	-	**15**

Note: Nucleotide sequence data reported in this paper are available in the GenBank databases under the accession numbers: KM454141 to KM454156

## Discussion

CL due to *L*. *tropica* occurs in many endemic areas, especially North Africa, Middle East, Central and South Asia. *L*. *tropica* has not been isolated from *P*. *sergenti* frequently [39, 40] as reported here. The transmission cycles of *L*. *tropica* vary with different geographic locations and generally have not been known to require a sylvatic reservoir. One noticeable exception is the atypical focus in Northern Israel, where *L*. *tropica* is transmitted by *P*. (*Adlerius*) *arabicus* with hyraxes as the reservoir, adjacent to a classical anthroponotic focus [41].

A high biodiversity of sand flies was observed in the region under study, representing 57% (13/23) of all *Phlebotomus* species hitherto described in Morocco. We have demonstrated that *P*. *sergenti* was the predominant vector species (45.75%) present abundantly throughout the collection period.

In agreement with previous reports, our findings showed that the rate of *L*. *tropica* infection is higher in fed compared with unfed females, as the latter include largely newly emerged adults, which are expected to contain no *L*. *tropica* before taking blood meals [42]. Our overall rate of infection with *L*. *tropica* is as high as 5.69%, increasing from 2.95% in June to 10.79–13.09% in September-October. These data are consistent with the previous report in the period of high transmission during fall season [43]. On the other hand, the rate of infection found in this area was much higher than the 1.44–3.66% found in other emerging foci in Morocco [44, 45].

The prevalence of infection seen in the study area may result from the increase in the circulation of *L*. *tropica* to a high level. Indeed, the number of CL cases has increased steadily, reaching a hundred per year since the first outbreak of CL in 2006 [46]. Our results confirm that *P*. *sergenti* is most likely the primary and the only vector of *L*. *tropica* in this region, not only due to the fact that it was the only species found infected with *L*. *tropica* but also because of its high abundance and infection rate. In Morocco, *L*. *tropica* was first detected in *P*. *sergenti* sand flies over 30 years ago [5] and more recently in emerging CL foci in the Centre and the North of Morocco [45, 47].

Species of the sub-genus *Larroussius* constitute more than 41% of sand flies collected in the studied area with *P*. *perniciosus* being the most abundant. Members of sub-genus *Larroussius* are known to be the vectors of *L*. *infantum*. However, PCR screening revealed no *Leishmania* infection in species belonging to this sand fly sub-genus.

Molecular methods are useful for the detection and characterization of parasites directly in field-collected samples without culturing. Previous evidence indicates that cultivation selects subpopulations of the parasites in the biological samples, including the emergence of nonpathogenic trypanosomatids [48]. In addition, direct analysis of *Leishmania* DNA in field collected samples can produce unexpected findings, including co-infections by more than one *Leishmania* species in a single subject [49]. In the present study, PCR-based amplification of the ITS1-5.8S rRNA gene-ITS2 proved to be highly sensitive for identifying *Leishmania* in sand flies to the species and strain levels [28, 47]. Analysis of ITS1-5.8S rRNA gene-ITS2 sequences from 31 *P*. *sergenti* specimens makes it possible to show great heterogeneity of *L*. *tropica*, segregating them into 16 haplotypes and revealing their phylogenetic relatedness to Indian strains (FJ948458, FJ948456, FJ948465 and FJ948455) and one Moroccan strain isolated from CL patient (KC145159).

*L*. *tropica* is a very heterogeneous species and a high degree of intraspecific polymorphism has been described based on isoenzyme analysis and other molecular methods [50–52]. Morocco is known for being the country with the highest number of *L*. *tropica* zymodemes described so far: 8 zymodemes were characterized from human, dogs and the sand flies, e.g. *P*. *sergenti* [5, 11]. On the other hand, *L*. *tropica* strains from *P*. *sergenti* showed the largest range of zymodemes compared to isolates from the vertebrate hosts [12]. Our results underscore the high polymorphism of *L*. *tropica* detected in *P*. *sergenti*.

Furthermore, sequence analysis revealed seven sequences with two superimposed peaks at a single nucleotide position. There are at least three possible interpretations for this: (i) sequence heterogeneity of different copies in ITS1-5.8S rRNA gene-ITS2 organized as tandem repeats in the *Leishmania* genome; (ii) sequence heterogeneity of the DNAs in different individual cells, since our samples were amplified from a cell population, which can be heterogeneous in a field collected sample; (iii) these allelic base substitutions are suggestive of heterozygosity of *L*. *tropica*, indicating possible sexual reproduction in *L*. *tropica*. To confirm the last hypothesis, the two first possibilities must be ruled out by sequencing genes known to be single-copy per haploid genome and by amplifying single-copy genes from a single cell or a population grown up from a single cell. Hybrid marker profiles detected in field isolates have been considered as evidence for sexual recombination in *Leishmania* [53–55], even if the principal reproductive mechanism in *Leishmania* is asexual via clonal reproduction. Rogers *et al*. (2014) used whole genome sequencing to investigate genetic variation in *L*. *infantum* parasites isolated from naturally infected sand flies. They showed, for the first time, that variation in these parasites arose following a single cross between two diverse strains and subsequent recombination, despite mainly clonal reproduction in the parasite population. Their results are the most direct evidence of sexual recombination in a natural population of *Leishmania* [56].

Although cutaneous leishmaniasis caused by *L*. *tropica* is usually considered an anthroponotic infection [57], zoonotic transmission was reported in Jordan, the Palestinian Authority and Israel [58, 59]. In the present rural CL focus, zoonotic transmission may occur, as the human cases of CL are sporadic and the major vector species feeds not exclusively on humans. *P*. *sergenti* was found in high densities inside houses and animal shelters, which indicates a very close contact between this species and humans and domestic animals. The results of the blood meal analyses showed the important relationship between *P*. *sergenti* populations and humans, since 41 *P*. *sergenti* (64%) fed on humans. Chicken constituted the second highest source of blood taken by this vector (12.5%), however chickens are not susceptible to *Leishmania* infection, because of some physiological characteristics, including their body temperature of 41.0°C. Furthermore, infected sand flies may eliminate *Leishmania* parasite, when they take a second blood meal from chickens or birds [60, 61].

Among 7 *P*. *sergenti* females feeding on rodents, one specimen was found to be infected by *L*. *tropica*, which make rodents a suspected reservoir of this *Leishmania* species. Indeed, several rodent species are assumed to have a role in transmission of *L*. *tropica* [58, 59, 62, 63]. *P*. *sergenti* was found also to feed on wild animals such as monkeys and bats suggestive of their potential role as a reservoir for further investigation.

### Conclusion

In the present study, we demonstrated a high infection rate of *L*. *tropica* in a single *Phlebotomus* species, that predominantly, but not exclusively, feeds on humans. This confirms the status of *P*. *sergenti* as the primary vector of *L*. *tropica* in this focus. Our results highlighted the high diversity of *L*. *tropica* in Morocco. A zoonotic transmission of *L*. *tropica* in this region is further suggested from the blood meal analysis of the vector. The potential for an animal reservoir host for this species awaits further exploration.
